# Unequal burden of Zika-associated microcephaly among populations with public and private healthcare in Salvador, Brazil

**DOI:** 10.1016/j.ijid.2022.04.030

**Published:** 2022-07

**Authors:** Adeolu Aromolaran, Katiaci Araujo, Joseph B. Ladines-Lim, Nivison Nery, Mateus S. do Rosário, Valmir N. Rastely, Gracinda Archanjo, Dina Daltro, Gustavo Baltazar da Silveira Carvalho, Kleber Pimentel, João Ricardo Maltez de Almeida, Isadora Cristina de Siqueira, Hugo C. Ribeiro, Jamary Oliveira-Filho, Daiana de Oliveira, Daniele F. Henriques, Sueli G. Rodrigues, Pedro F. da Costa Vasconcelos, Antonio R.P. de Almeida, Gielson A. Sacramento, Jaqueline S. Cruz, Manoel Sarno, Bruno de Paula Freitas, Adriana Mattos, Ricardo Khouri, Mitermayer G. Reis, Albert I. Ko, Federico Costa

**Affiliations:** 1 Yale School of Public Health, New Haven, USA; 2 Hospital Aliança, Salvador, Brazil; 3 Fundação Oswaldo Cruz, Salvador, Brazil; 4 Hospital Geral Roberto Santos, Secretária da Saúde do Estado da Bahia, Salvador, Brazil; 5 Escola Paulista de Medicina, esc, São Paulo, Brazil; 6 Harris Birthright Center for Fetal Medicine, King's College Hospital, London, UK; 7 Programa de Pos-Graduacao em Ciencias da Saude, Faculdade de Medicina da Bahia, Universidade Federal da Bahia, Salvador, Brazil; 8 Faculdade de Medicina da Bahia, Instituto da Saúde Coletiva and Hospital Universitário 8Professor Edgard Santos, Universidade Federal da Bahia, Salvador, Brazil; 9 Instituto Evandro Chagas, Ministério da Saúde, Pará, Brazil; 10 Universidade Federal da Bahia, Salvador, Brazil

**Keywords:** Zika Virus, Microcephaly, Fetus, Socioeconomic Status

## Abstract

**Objectives:**

To describe the differences in clinical presentation and relative disease burden of congenital Zika syndrome (CZS)-associated microcephaly at 2 large hospitals in Salvador, Brazil that serve patients of different socioeconomic status (SES).

**Methods:**

Clinical and serologic data were collected prospectively from pregnant women and their infants, who delivered at 2 study centers during the 2015–2016 Zika virus (ZIKV) epidemic in Salvador, Brazil.

**Results:**

Pregnant women from Salvador, Brazil delivering in a low SES hospital had 3 times higher ZIKV exposure rate than women at a high SES hospital. However, different SES hospitals had similar prevalence of infants with CZS-associated microcephaly (10% vs 6%, p = 0.16) after controlling for ZIKV exposure in their mothers.

**Conclusions:**

Our study supports the positive association between low SES, high maternal ZIKV exposure, and high rates of CZS-associated microcephaly.

## Highlights

• In-utero Zika exposure causes severe birth defects known as Congenital Zika Syndrome
• The outbreak of Congenital Zika Syndrome was related to maternal Zika virus exposure
• Socioeconomic status plays a role in Zika virus exposure.

## 1 Introduction

**Figure 1:**
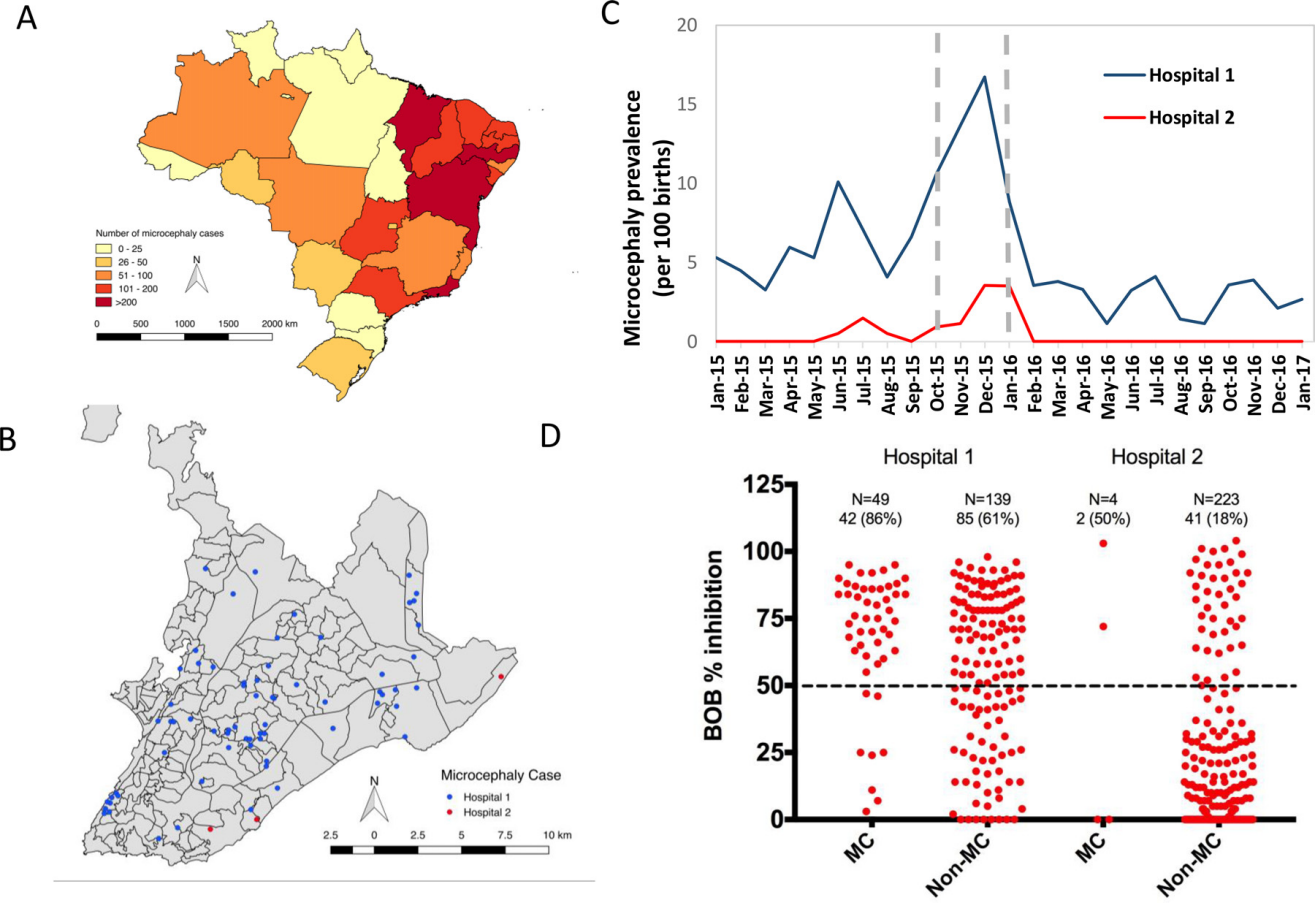
**Prevalence and distribution of microcephaly and ZIKV exposure between January 2015 and January 2017**. Shown are the distribution of new microcephaly cases in Brazil in 2015 (A). Municipalities of residence within the Salvador metropolitan region for infants with microcephaly born at the study hospitals are also shown (B). Monthly prevalence of microcephaly at the study hospitals are shown (C) with gray dotted lines outlining the period of peak prevalence. BOB inhibition titers for the 188 mothers tested in Hospital 1 and 227 mothers tested in Hospital 2 are plotted by microcephaly status (D).

In utero exposure to Zika virus (ZIKV) is an important cause of microcephaly and syndromic congenital defects worldwide (Weaver et al., 2016). The largest outbreak of congenital Zika syndrome (CZS)-associated microcephaly in Brazil occurred between 2015 and 2016 and disproportionately impacted states in the northeast region, which has among the lowest household incomes in the country (Figure 1A) (Coelho and Crovella, 2017). A serologic surveillance study in 1 northeastern state, Bahia, showed a significantly higher prevalence of ZIKV exposure in neighborhoods with lower income, lower levels of education, and poorer housing, suggesting that low socioeconomic indicators lead to more environmental exposure to ZIKV (Netto et al., 2017). Previous ecological studies on the basis of passive surveillance have also suggested an association between low socioeconomic status (SES) and ZIKV exposure (Ali et al., 2017). A retrospective case control study found an association between non-White ethnicity and odds of microcephaly but failed to find an association with other SES indicators (de Araújo et al., 2018). There is a lack of high-quality prospective studies evaluating the association between SES indicators and clinical outcomes related to ZIKV, such as CZS-associated microcephaly.

In this study, we evaluate the burden and clinical presentation of CZS-associated microcephaly at 2 different hospitals that serve populations of different SES.

## 2 Methods

We conducted a prospective cohort study at 2 tertiary referral hospitals in Salvador, Brazil (Figure 1A): Hospital 1 is a large public state hospital and Hospital 2 is a large private hospital (Figure 1B). We obtained pertinent anthropometric information for all infants born during the period between January 1, 2015 and January 31, 2017 to identify temporal changes in the incidence of microcephaly. We defined cases of microcephaly and severe microcephaly as newborns with a head circumference more than 2 and 3 SDs below the mean of the Intergrowth-21st standard, respectively. Analysis of microcephaly data revealed a peak in prevalence between October 1, 2015 and January 31, 2016 (Figure 1C). We geolocated the residence of microcephaly cases. Cases in the same census track were aggregated and color-coded into the map (Figure 1A-B) using Quantum Geographic Information System (Q-GIS) (Team, 2017).

Infants with microcephaly and their mothers were enrolled as a pair. They had biological samples (serum, peripheral blood, or cord blood) collected for diagnostic ZIKV testing. Serological testing was additionally conducted on a control group of infants without microcephaly (Supplemental Figure 1). Infant blood was tested using an in-house IgM ELISA (Martin et al., 2000). Previous maternal exposure to ZIKV was defined by presence of ZIKV IgG, as evidenced by positive NS1 blockade-of-binding (BOB) assay results on serum testing, which has a reported sensitivity of 91.8%–95% and a specificity of 88.9%–95.9% (Balmaseda et al., 2017). All infants with microcephaly were evaluated by a pediatric neurologist and a pediatric ophthalmologist and were referred for neuroimaging studies (CT, MRI, or transcranial ultrasound) as clinically indicated. Clinical assessments were performed between 0–4 months after birth. Infants who did not have anthropometric data available were not included in this analysis. CZS was defined as microcephaly and at least 1 of the following: characteristic findings on neuroimaging studies as described by Aragao et al (2016); features consistent with fetal brain disruption sequence such as overlapping cranial sutures and occipital skin fold; ophthalmologic abnormalities such as macular scarring, chorioretinal atrophy, and other structural abnormalities; and/or axial or appendicular hypertonia or congenital contractures such as clubfoot or arthrogryposis (de Fatima Vasco Aragao et al., 2016; de Paula Freitas et al., 2016). Demographic data, clinical characteristics, and serologic characteristics were compared for infants and their mothers across hospitals for statistical differences. Continuous variables were compared using the Mann-Whitney *U* test, whereas categorical variables were compared using the Fisher exact test. A 2-tailed p-value of < 0.05 was considered significant.

## 3 Results

In this study, we obtained information from 690 of 927 (74%) eligible mothers in Hospital 1 and 757 of 767 (99%) eligible mothers in Hospital 2. We found a striking difference in rates of caesarean delivery between the 2 hospitals (51% vs 83%, p <0.001; Table 1), which is consistent with previous studies. It is thought to be due to nonclinical factors related to the care of pregnant women with higher SES such as physician attitudes, availability of services, and maternal age (Ribeiro et al., 2007). Mothers in Hospital 1 were younger than those in Hospital 2 (Table 1). Despite similar geographic distribution, our findings show markedly higher rates of ZIKV exposure, as evidenced by ELISA positive results, in women who delivered at Hospital 1 (64% vs 19%, p <0.001) (Figure 1D).

**Table 1:** Characteristics of mothers and infants between October 1, 2015 and January 31, 2016

Characteristic	Hospital 1	Hospital 2	
	N=927	N=767	
	*n*/total no. (%) or median (IQR)	*n*/total no. (%) or median (IQR)	p-value
**Hospital**^a^			
Patients with public health insurance (%)	100%	0%	**<0.001**
Total beds (No.)	640	213	–
Maternity beds (No.)	90	15	–
Neonatal ICU beds (No.)	17	15	–
Births (No.)	2684	1850	–
Caesarean sections (%)	51%	83%	**<0.001**
**Mothers**			
Recruited mothers^b^	690/927 (74)	757/767 (99)	**<0.001**
Age (y)	26 (21-32)	33 (30-36)	**<0.001**
Metropolitan area	535/690 (78)	589/757 (78)	0.94
Median neighborhood income ($)^c^	273	1125	**<0.001**
ZIKV IgG positive at delivery	127/188 (68)	43/227 (19)	**<0.001**
Prevalence (95% CI)^d^	64 (61-67)	19 (16-22)	**<0.001**
**Newborn infants**			
Recruited infants^e^	732/927 (79)	764/767 (100)	**<0.001**
Gestational age (weeks)	39 (37-40)	39 (38-39)	0.88
Female	344/732 (47)	346/764 (45)	0.45
Microcephaly	83/732 (11)	8/764 (1)	**<0.001**
Adjusted prevalence (95% CI)^f^	11 (9-14)	1 (0-2)	**<0.001**
Microcephaly among infants of mothers with positive NS1 BOB at delivery	42/127 (33)	2/43 (5)	**<0.001**
Prevalence (95% CI) d	18 (14-28)	6 (3-11)	**<0.001**
CZS-associated microcephaly	46/732 (6)	4/764 (0.5)	**<0.001**
Prevalence (95% CI)^f^	8 (6-10)	1 (0-2)	**0.04**
CZS-associated microcephaly among mothers with positive NS1 BOB at delivery	35/127 (28)	2/43 (5)	**<0.001**
Prevalence (95% CI) d	10 (8-13)	6 (3-11)	0.16

a Hospital data shown from January 1, 2015 – December 31, 2015.

b Mothers for which age and residence was available

c Financial data (USD) available for 484 mothers in Hospital 1 and 108 mothers in Hospital 2

d ZIKV IgG prevalence was sample adjusted to equally weigh microcephaly and non-microcephaly arms

e Infants for which all anthropometric data was available

f Microcephaly prevalence adjusted to account for unit non-responders

Infant anthropometric data was available for 732 (79%) infants in Hospital 1 and 764 (100%) infants from Hospital 2. The prevalence of microcephaly was higher in Hospital 1 (11% vs 1%, p <0.001) (Table 1, Figure 1C).

Of the infants with microcephaly, 66 cases in Hospital 1 and 4 cases in Hospital 2 completed a full workup to assess for clinical evidence of CZS-associated microcephaly. Infants who underwent partial workup showed no manifestation of CZS-associated microcephaly; however, a definitive diagnosis could not be made without a full assessment. Of the 66 infants who received a full workup, 46 (70%) in Hospital 1 and 4 (100%) in Hospital 2 fit clinical criteria for CZS-associated microcephaly (p = 0.32) (Supplemental Table 1). Among mothers with ZIKV exposure, we found a 10% prevalence of CZS-associated microcephaly in Hospital 1 and 6% in Hospital 2 (p = 0.16).

## 4 Conclusions

Our study hospitals experienced a 3-fold rise in prevalence of microcephaly during the outbreak, and the outcomes for these infants were uniformly severe. We found that mothers from Hospital 1 with lower SES have higher ZIKV exposure rates as well as a higher prevalence of infants with microcephaly.

Our innovative study compares the prevalence of microcephaly among infants of mothers from hospitals with different socioeconomic profiles. Hospital 1 only serves patients with public health insurance, which includes patients from the lowest socioeconomic divisions. These mothers live in neighborhoods with 75% lower median household income compared to Hospital 2 (p <0.001). Hospital 1 experienced a much higher prevalence of microcephaly than Hospital 2. The rate of microcephaly was as high as 11% in Hospital 1, whereas Hospital 2 experienced a rate of 1%, which is similar to the national rates cited in previous studies (Magalhães-Barbosa et al., 2016). Importantly, infants with microcephaly in both hospitals show similar clinical characteristics of CZS-associated microcephaly, with the most prevalent symptoms being intracranial abnormalities, severe microcephaly, and axial and appendicular hypertonia (Supplemental Table 1).

The rate of ZIKV exposure was 3 times higher among mothers in Hospital 1 despite both groups of mothers living in the Salvador metropolitan area. In fact, ZIKV exposure level in Hospital 1 was similar to that observed in residents of slum communities in the same city (64% and 73%, respectively) (Rodriguez-Barraquer et al., 2019). After controlling for maternal ZIKV exposure, we found that rates of CZS-associated microcephaly were similar between both hospitals. This suggests that there are socioeconomic cofactors influencing maternal ZIKV exposure, which in turn influences development of CZS-associated microcephaly among infants.

Our study has important limitations. We studied hospital populations and used hospital characteristics to make inferences about the SES of mothers. These inferences cannot be reliably extended to individual mothers in the group. Additionally, our clinical screening only accounts for CZS-associated microcephaly cases with the most severe outcomes. Infants may have milder presentations that will manifest later in childhood and thus, we may be underestimating the prevalence of CZS (Ventura et al., 2016). We also were not able to recover data from every birth, especially in Hospital 1. We believe the missing data are random and likely reflects our overall population; however, follow-up studies are needed.

Our study supports the link between low SES, high maternal ZIKV exposure, and high rates of CZS-associated microcephaly. Interestingly, microcephaly rates are not different when only accounting for mothers exposed to ZIKV. Together, these results suggest that low SES is associated with an increased rate of maternal ZIKV exposure, which in turn is associated with risk of CZS.

## Ethical Approval

This study was approved by the institutional review boards of Roberto Santos General Hospital (No. 1.422.021) and Yale University (1.422.021). The authors attest to the accuracy and completeness of data as well as the fidelity of the study to the protocol.

## Funding Source

This study was supported by Oswaldo Cruz Foundation; Secretariat of Health Surveillance; Brazilian Ministry of Health; Wellcome Trust, Grant/Award Number: 102330/Z/13/Z; NSF-NIH, Grant/Award Number: 5 R01 AI052473, 5 U01 AI088752, 1 R25 TW009338, 1 R01 AI121207, F31 AI114245, R01 AI052473, U01 AI088752, R01 TW009504, and R25 TW009338. Fogarty International Center (R25 TW009338). Fundação de Amparo à Pesquisa do Estado de São Paulo (FAPESP) projects 2016/08727-5 and National Council for Scientific and Technological Development – CNPq. Fundação de Amparo à Pesquisa do Estado da Bahia (FAPESB) projeto (PET0021/2016). The study sponsors had no role in study design, data collection, data analysis, or manuscript writing.

## Declaration of Competing Interest

The authors declare that they have no known competing financial interests or personal relationships that could have appeared to influence the work reported in this paper.

## Appendix Supplementary materials

Image, application 1
