# Supplementary material for: Unequal burden of Zika-associated microcephaly among populations with public and private healthcare in Salvador, Brazil
Source: Int J Infect Dis. 2022 Jul;120:201–4. doi: 10.1016/j.ijid.2022.04.030 (PMC9119857; doi:10.1016/j.ijid.2022.04.030)
Supplement: Supplementary file 1 [file mmc1.docx]

**SUPPLEMENTAL METHODS**

## Serologic confirmation of Zika exposure in mothers and infants

To estimate the prevalence of ZIKV exposure among all mothers and infants born during the study period, we invited a cohort of infants without microcephaly and their mothers to participate in a serological survey. During the active surveillance period from December 19, 2015 to January 31, 2016, we collected infant blood samples and maternal serum samples from the first five births without microcephaly each day at hospitals 1 and 2. ZIKV infection in infants was defined by a positive IgM ELISA result as defined above. Prior ZIKV infection in mothers was defined by positive NS1 Blockade-of-Binding **(**BOB) assay results on serum testing. BOB assay was conducted according to previously described protocol, using the same antigens, primers and incubation times ([Balmaseda et al., 2017](#_ENREF_3)). After incubation with ZKA35 antibodies, plates were read in an ELISA reader at 405 nm. The percentage of inhibition was calculated as follows: [1 − (OD sample − OD negative control)/(OD positive control − OD negative control)] × 100. BOB assay was considered positive if inhibition was 50% or greater.

## Prevalence of microcephaly, CZS and maternal ZIKV exposure

Crude prevalence of maternal ZIKV exposure was calculated for each hospital using the following formula:

$$Crude prevalence \left( \% \right)=\frac{Total number BOB positive}{Total number tested}x 100$$

Prevalence of maternal exposure was then adjusted to account for differential sampling among mothers of microcephaly and infants without microcephaly as shown below. To achieve this, maternal exposure was weighted according to microcephaly status of the infant and the proportion of the total number of cases that was sampled. We adjusted prevalence using sampling weights to calculate ZIKV exposure estimates that are representative of the population of mothers in our study.

$$Adjusted prevalence \left( \% \right)=$$

$$\frac{\left[ \left( \frac{\# of MC positive, BOB positive}{\# of MC tested}xTotal \# of MC \right)+\left( \frac{\# of MC negative, BOB positive}{\# of non MC tested}xTotal \# ofnon MC \right) \right]}{Total number of mothers}x 100$$

We estimated the prevalence of microcephaly and CZS among ZIKV exposed mothers by calculating the ratio of microcephaly/CZS prevalence to the adjusted prevalence of maternal ZIKV exposure.

The central limit theorem was used to calculate binomial confidence intervals for prevalence of microcephaly and maternal ZIKV exposure when the number of positive observations was greater than 5. For ratios in which the number of positive observations was 5 or fewer, the Wilson score interval was used to calculate the binomial confidence interval ([Wilson, 1927](#_ENREF_16)).When calculating the ratio of prevalence of microcephaly to prevalence of maternal ZIKV exposure, a ratio of two binomial proportions, Koopman’s method was used to calculate the confidence interval ([Koopman, 1984](#_ENREF_7)).


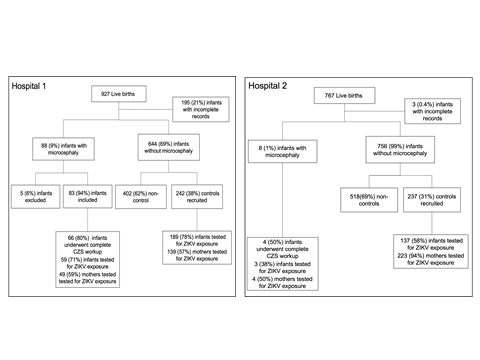


**Supplemental Figure 1. Prospective cohort recruitment and testing between October 1, 2015 and January 31, 2016.** Shown are the number of live births in each hospital as well as the number of infants with microcephaly, without microcephaly and with missing anthropometric data. Among infants with microcephaly in Hospital 1, 5 were excluded as they had anencephaly, encephalocele or phenotype of another well-defined congenital syndrome. Numbers of infants and mothers that completed workup are shown. Among infants without microcephaly, a cohort of infants and mothers were recruited as controls and the number of mothers and infants that followed up for workup are shown.

**Supplemental Table 1.** Clinical characteristics and laboratory evaluation of microcephaly cases during epidemic period between October 1, 2015 to January 31, 2016.

| Characteristic | Hospital 1 | | Hospital 2 | |  |
| --- | --- | --- | --- | --- | --- |
|  | N = 83 | | N = 8 | |  |
|  | No. tested | No. (%) or median (IQR) | No. tested | No. (%) or median (IQR) | p-value |
| **Maternal Characteristics** |  |  |  |  |  |
| Age (y) | 83 | 23 (20-29) | 7 | 32 (30-33) | **<0.001** |
| Gravidity^a^ | 51 | 2 (1-3) | 5 | 2 (2-2) | 0.47 |
| Parity^a^ | 51 | 2 (1-3) | 5 | 2 (1-2) | 0.40 |
| **Infant Characteristics** |  |  |  |  |  |
| Gestational age (weeks) | 83 | 39 (38-40) | 8 | 39 (38-39) | 0.92 |
| Caesarean delivery | 83 | 46 (59) | 6 | 4 (67) | 0.69 |
| Female gender | 83 | 40 (48) | 8 | 4 (50) | 1.00 |
| **Anthropometrics** |  |  |  |  |  |
| Head circumference (cm) | 83 | 30 (28-31) | 8 | 29 (28-31) | 0.72 |
| Percentiles | 83 | 0.2 (0.0-1.6) | 8 | 0.0 (0.0-1.3) | 0.52 |
| Severe microcephaly^b^ | 83 | 39 (47) | 8 | 5 (63) | 0.48 |
| Disproportionate microcephaly^c^ | 83 | 66 (80) | 8 | 8 (100) | 0.34 |
| Weight (kg) | 83 | 2.5 (2.2-2.9) | 8 | 2.7 (2.3-3.1) | 0.40 |
| Percentile | 83 | 12 (2.6-29) | 8 | 14 (4.2-46) | 0.57 |
| Small for gestational age^d^ | 83 | 38 (46) | 8 | 4 (50) | 1.00 |
| Length (cm) | 83 | 47 (45-49) | 8 | 47 (46-48) | 0.98 |
| Percentile | 83 | 22 (2.6-54) | 8 | 10 (4.6-41) | 0.79 |
| **Clinical manifestations** |  |  |  |  |  |
| Craniofacial dysmorphism^e^ | 83 | 20 (24) | 5 | 1 (20) | 1.00 |
| Congenital contractures |  |  |  |  |  |
| Arthrogryposis | 83 | 8 (10) | 7 | 1 (14) | 0.53 |
| Talipes equinovarus | 83 | 5 (6) | 7 | 1 (14) | 0.39 |
| Axial and/or appendicular hypertonia | 78 | 39 (50) | 5 | 0 (0) | 0.06 |
| Ocular lesions^f^ | 64 | 23 (36) | 0 | 0 | -- |
| Intracranial abnormalities on imaging | 52 | 43 (83) | 4 | 4 (100) | 1.00 |
| Ventriculomegaly | 52 | 38 (73) | 4 | 4 (100) | 0.56 |
| Calcifications | 52 | 23 (44) | 4 | 3 (75) | 0.33 |
| Criteria for CZS^g^ | 66 | 46 (70) | 4 | 4 (100) | 0.32 |
| **Laboratory evaluation** |  |  |  |  |  |
| Laboratory-confirmed ZIKV exposure | 59 | 27 (46) | 3 | 1 (33) | 1.00 |
| IgM in cord blood | 55 | 24 (44) | 2 | 1 (33) | 1.00 |
| PCR positive | 55 | 6 (11) | 3 | 1 (33) | 0.33 |

^a^ Information includes the newborn infant case.

^b^ Head circumference <-3 SD of the InterGrowth-21st reference.

^c^ Head circumference <3rd percentile and weight or length >3rd percentile of the InterGrowth-21st reference.

^d^ Weight <10th percentile of the InterGrowth-21st reference.

^e^ Includes findings of partially collapsed skull, overlapping cranial sutures and prominent occipital bone.

**^f^** Includes findings of optic nerve hypoplasia, pallor and increased cusp-disc ratio.

^g^ CZS criteria is microcephaly in addition to one of the following: hypertonia, abnormal neuroimaging, congenital contractures, abnormal eye exam and craniofacial abnormalities.
